# Aneurism of the Aorta

**Published:** 1855-12

**Authors:** John L. Hostetter

**Affiliations:** Mt. Carroll


ART. II.—*Aneurism of the Aorta.*

THE following letter was kindly furnished to us by Professor BRAINARD, and will be read with interest.—ED.

MT. CARROLL, Oct. 20th, 1855

DR. BRAINARD:

DEAR SIR,—The following case may not be uninteresting to you.

On the 6th of August, the present year, I was called to see John Pyle, aged 73 years; attacked with bilious colic, which yielded speedily under hydrarg. sub. murias and opiates, followed with purgatives. In a few days he was able to sit on the arm-chair for two and three hours at a time. My visits were continued, however at his request, and about the tenth day I discovered a tumor the size of a hen's egg on the left side of the umbilicus, a little below, having a strong pulsation. Suspecting an aneurism of the aorta, I had my friend and fellow practitioner of this town called in, (Dr. B. P. Miller), who agreed with me in the diagnosis. He recommended an abstemious diet; perfect rest, and ordered such indirect depletives, as would lessen the circulation, at the same time assuring our patient, that he could not hope for an ultimate recovery. About this time, there was an aching of the thigh and back; along the course of the sacrosciatic nerve, which could only be relieved by chloroform.

About the 13th September, he could only find relief by inhaling chloroform. The tumor had now enlarged to the size of a child's head; beating more furiously than ever. At this juncture some of the friends advised sending for a certain Dr. Reed, who pronounced it disease of the liver. He administered something which caused retching and frequent straining attempts at vomiting until September 30th, when I was again summoned in haste, to find my patient pulseless and worn out.

This morning, Sept. 30th, the sac of the aneurism had evidently bursted, but by keeping the patient quiet the coagulated blood around the orifice of the rupture had arrested the hemorrhage.

The same evening the liver Dr. Reed made his *debut* again, and assured the patient and his friends again that he would "have him about in a few days."

On Tuesday morning he died, and according to his request I made a post mortem examination, in presence of my brother, Dr. A. Hostetter, and Dr. J. Pennybaker, and likewise the friends of the deceased. On laying open the parieties of the abdomen, a large tumor the size of a man's head presented itself with a rupture on the left side near superior part. It had displaced the intestines and occupied the greater portion of the left iliac, and umbilical region. The grumous blood emptied into the cavity of the abdomen, having been removed the sac was opened, and about four or five pounds of coagulated blood taken out.

Within this sac numerous ossifications were discovered, two of which find enclosed. The tumor or sac embraced about two inches of the left primitive iliac, and about five inches of the aorta. The right iliac seemed sound with the exception of ossified portions distinctly perceptible.

About two months before the attack of bilious colic, before alluded to, the patient jumping over a fence, felt "something giving way" in his left side.

The liver, stomach, spleen, and intestines were normal, or in fact presented the very picture of a healthy state.

Very Respectfully,

JOHN L. HOSTETTER.

